# DNMT3a and TET2 in adipocyte insulin sensitivity

**DOI:** 10.18632/oncotarget.26246

**Published:** 2018-10-19

**Authors:** Sneha Damal Villivalam, Jinse Kim, Sona Kang

**Affiliations:** Nutritional Sciences and Toxicology Department, UC Berkeley, Berkeley, CA, USA

**Keywords:** DNA methylation, epigenetics, diabetes, insulin resistance, adipocytes

Insulin resistance (IR) is a key pathogenic feature of type 2 diabetes and occurs in a wide array of other maladies including obesity, aging, cardiovascular disease, and certain types of cancer. It results from an intricate interaction between genetic make-up and environment, suggesting it's orchestrated by epigenetic mechanisms. In fact, a plethora of studies have found that changes in DNA methylation are associated with metabolic dysregulation [1, 2], but methylation's functional role is poorly understood.

DNA methylation is an epigenetic mark involving the covalent transfer of a methyl group to the C-5 position (5mC) of cytosine by DNA methyltransferases (DNMTs). The initial finding that DNMT levels are significantly increased in diet-induced obesity and genetically obese *ob/ob* mice led us to postulate that it plays a large role in IR [3]. We found that DNMT3a, in particular, is both necessary and sufficient to mediate IR in cultured mouse and human adipocytes. Indeed, adipose-specific Dnmt3a-knockout mice are protected from diet-induced IR and glucose intolerance, with no change in their body weight or composition. Through RNA-seq studies, we found that an important downstream target is *Fgf21*, which is known to facilitate glucose uptake in adipocytes. In patients with diabetes, DNA methylation at the *FGF21* locus is elevated in association with decreased expression of *FGF21* in adipose tissue. Importantly, FGF21 expression partially rescues Dnmt3a-mediated IR *in vitro*, indicating that it is helping mediate the effect of DNMT3a on IR.

DNMTs methylate DNA, but this methylation can be erased by the TET proteins (TET1, 2, and 3), which oxidize 5mC to hydroxymethylcytosine (5hmC), which is then converted to unmethylated cytosine (5C) through base excision repair (BER) and thymidine DNA glycosylase (TDG) [4]. Given the functional role of DNMT3a in the development of IR, we hypothesized that the TET proteins play a counter-regulatory role. Indeed, adipose expression of TET2 is significantly decreased in diet-induced IR [5], and TET2 gain-of-function promotes insulin sensitivity while loss-of-function is necessary for insulin sensitization of PPARγ agonist, Rosiglitazone (Rosi). TET2 is required for Rosi-dependent gene activation of certain PPARγ targets, which is accompanied by changes in the DNA demethylation profile at their promoter regions (Figure 1). Furthermore, TET2 physically interacts with PPARγ to sustain PPARγ binding to target *loci* upon PPARγ activation with Rosi (Figure 1). Together, these studies suggest that TET2 facilitates the transcriptional activity and insulin-sensitizing efficacy of PPARγ.

**Figure 1:**
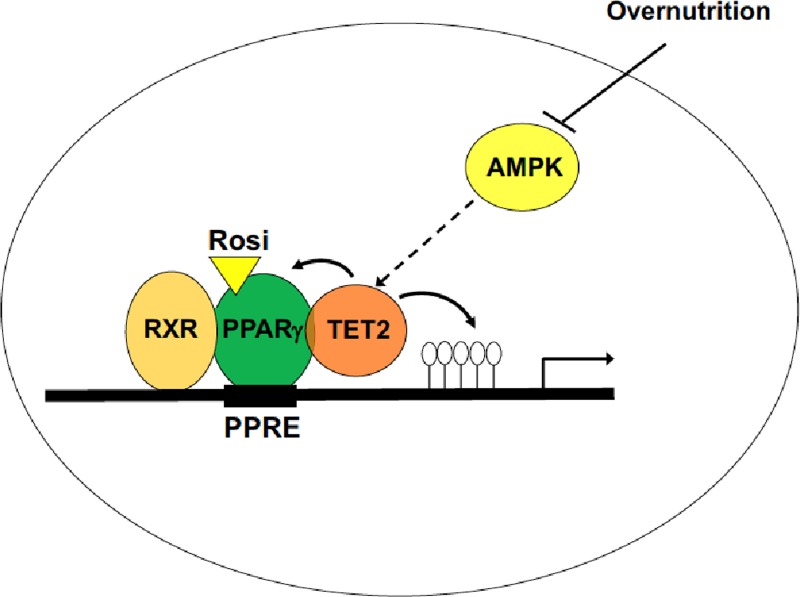
Proposed model of TET2 as a regulator of PPARγ-dependent transcription in adipocytes TET2 physically interacts with PPARγ to sustain PPARγ binding at PPARγ responsive elements (PPREs) and to facilitate the transcriptional activation of PPARγ in response to Rosiglitazone (Rosi). TET2 causes demethylation at the promoter regions of some PPARγ target genes such as *Adipoq* in a site-specific manner, which can affect insulin sensitivity. Based upon recent finding by Wu et al, it is postulated that AMPK may act as an upstream of TET2 in adipocytes (Open circle; demethylated CpG).

In line with these findings, Wu et al recently published work revealing a novel axis between TET2 and AMPK in the regulation of glucose homeostasis [6]. This study found that hyperglycemia destabilizes TET2 through inhibiting AMPK-mediated TET2 phosphorylation at Ser99, which leads to downregulation of global 5hmC levels in the blood of diabetic patients. Furthermore, hyperglycemia-promoted tumor growth was suppressed by TET2, and the anti-tumor effect of Metformin appears to require the AMPK-TET2-5hmC axis. Together, these studies suggest that TET2 is a critical epigenetic sensor/regulator of glucose in the cell. It will be of great importance to find out whether this regulatory loop can be found in adipose and other metabolic tissues in the context of obesity and diabetes.

Several important questions still remain: 1) Do DNMT3a and TET2 directly converge to regulate insulin sensitivity? They functionally oppose one another, but physical interaction between the two was not detectable by co-immunoprecipitation, and most of their gene targets do not overlap [3, 5]. 2) What is the *in vivo* role of TET2 in adipose and other tissues? Studies on TET2 were conducted using cultured adipocyte models, thus physiological validation using tissue-specific knockout and transgenic mouse models will be critical. It will be intriguing to investigate whether adipose-specific *Tet2*- knockout mice are refractory to Rosi-driven insulin sensitization. 3) How do DNMT3a and TET2 affect the adipose epigenome? Investigation into the DNA methylation activity of DNMT3a and TET2 has been limited to the promoter regions of key target genes, but gene bodies and enhancers may also be methylated, having different impacts on gene regulation depending on the function of the region and CpG density. To gain a more comprehensive understanding, global DNA methylation profiling studies will be necessary, ideally using *in vivo* models.

Answering these questions will lead to a more detailed understanding of the mechanisms of DNMT3a and TET2, which may lead to identifying novel targets for the treatment of IR and relevant human diseases.

## References

[1] Barres R (2011). Am J Clin Nutr.

[2] Davegårdh C (2018). Mol Metab.

[3] You D (2017). eLife.

[4] Benner C (2015). Proc Natl Acad Sci USA.

[5] Bian F (2018). Metab Clin Exp.

[6] Wu D (2018). Nature.

